# Potential Reach of mHealth Versus Traditional Mass Media for Prevention of Chronic Diseases: Evidence From a Nationally Representative Survey in a Middle-Income Country in Africa

**DOI:** 10.2196/jmir.5592

**Published:** 2016-05-20

**Authors:** Maryam Yepes, Jürgen Maurer, Barathi Viswanathan, Jude Gedeon, Pascal Bovet

**Affiliations:** ^1^ University Institute of Social and Preventive Medicine (IUMSP) Department of Biology and Medicine, Lausanne University Hospital University of Lausanne Lausanne Switzerland; ^2^ Departments of Econometrics and Political Economics University of Lausanne Lausanne Switzerland; ^3^ Ministry of Health Public Health Department Victoria Seychelles

**Keywords:** digital divide, mHealth, eHealth, mass media, mobile phone, noncommunicable diseases, short message service, email, internet access, developing countries, low- and middle-income countries, Africa

## Abstract

**Background:**

Public radio and television announcements have a long tradition in public health education. With the global rise of computer and mobile device ownership, short message service (SMS) and email-based health services (mHealth) are promising new tools for health promotion.

**Objective:**

Our objectives were to examine 1) self-reported exposure to programs related to noncommunicable diseases (NCDs) on national public television and radio during the 12 months preceding the survey (2013–2014), 2) current ownership of a mobile phone, smartphone, computer, or tablet, and use of the Internet, and 3) willingness of individuals to receive SMS or emails with information on health, with a focus on distribution of these variables across different demographic, socioeconomic status (SES), and NCD risk groups.

**Methods:**

We obtained data in a population survey of 1240 participants aged 25–64 years conducted in 2013–2014 in the Seychelles, a rapidly developing small island state in the African region. We administered a structured questionnaire and measured NCD risk factors. Univariate and multivariate analyses explored the relationships between outcomes and sociodemographic variables.

**Results:**

Of 1240 participants, 1037 (83.62%) reported exposure to NCD-related programs on public television, while a lower proportion of 740 adults (59.67%), reported exposure via public radio (*P* <.001). Exposure to NCD-related programs on public television was associated with older age (*P* <.001) and female sex (*P* <.001), but not with SES, while exposure to NCD-related programs on public radio was associated with older age (*P* <.001) and lower SES (*P* <.001). A total of 1156 (93.22%) owned a mobile phone and ownership was positively associated with female sex (*P* <.001), younger age (*P* <.001), and higher SES (*P* <.001). Only 396 adults (31.93%) owned a smartphone and 244 adults (19.67%) used their smartphone to access the Internet. A total of 1048 adults (84.51%) reported willingness to receive health-related SMS, which was positively associated with female sex (*P* <.001), younger age (*P* <.001), and higher SES (*P* <.001). Controlling for SES, exposure to NCD-related programs on public television or radio and willingness to receive health-related SMS were not independently associated with a person’s NCD risk.

**Conclusions:**

Broadcasting health programs through traditional mass media (national public radio and television) reached the majority of the population under study, including older adults and those in lower socioeconomic groups. With a high penetration of mobile phones and willingness to receive health-related SMS, mHealth presents an opportunity for health programs, especially when targeted SMS messages are intended for younger adults and those in higher socioeconomic groups. By contrast, due to reduced Internet access, email-based programs had a more limited reach for health promotion programs. These findings emphasize the different reach of interventions using SMS or email versus traditional mass media, according to demographic and socioeconomic categories, for health education programs in a developing country.

## Introduction

Evidence suggests that prevention and control of noncommunicable diseases (NCDs) is one of the major health challenges of the 21st century, including in low- and middle-income countries [1,2]. Multifaceted health education strategies combined with multisectoral policies aimed at promoting healthy behaviors are needed to reduce the burden of NCDs [1-4]. Before planning new NCD-related interventions to raise health awareness in the population, it is important to assess the reach of the existing mass media campaigns and to evaluate the potential audience of the planned additional interventions [4]. In this study, we assessed the reach of health education programs on national public television and radio in the Seychelles and the potential reach of short message service (SMS) or email-based interventions (mHealth) according to age, sex, socioeconomic status (SES), and health risk groups.

Health education through traditional mass media such as television and radio, when provided at a sufficiently high frequency, can promote healthy behaviors [5,6]. Advantages of health education campaigns through traditional mass media include a wide audience reach, an easily augmentable frequency of delivery, a high degree of control over content, and a relatively low cost per person exposed [5]. On the other hand, limitations include difficulties in capturing audiences’ attention in an increasingly cluttered media environment, the 1-way flow of information from providers to consumers, and a limited ability to offer target-specific messages to pre-identified audiences [6].

To outweigh some of the limitations of health education programs based on traditional mass media, a growing number of SMS or email-based (mHealth) interventions have been used in both developed and developing countries. The steady rise in ownership of mobile phone and other digital communication technologies has facilitated mobile-based interventions, including the provision of health information through mobile messaging and emails [7]. For example, the use of mHealth was highlighted as a key strategy to combat NCDs in developing countries at the 2011 United Nations high-level meeting on NCDs [1]. Pursing this strategy, telecommunication agencies and the World Health Organization (WHO) launched the initiative “Be He@lthy Be Mobile” in 2015, which aims to leverage mobile technology, in particular text messaging and related apps, to help combat the growing global burden of NCDs [8].

**Figure 1:**
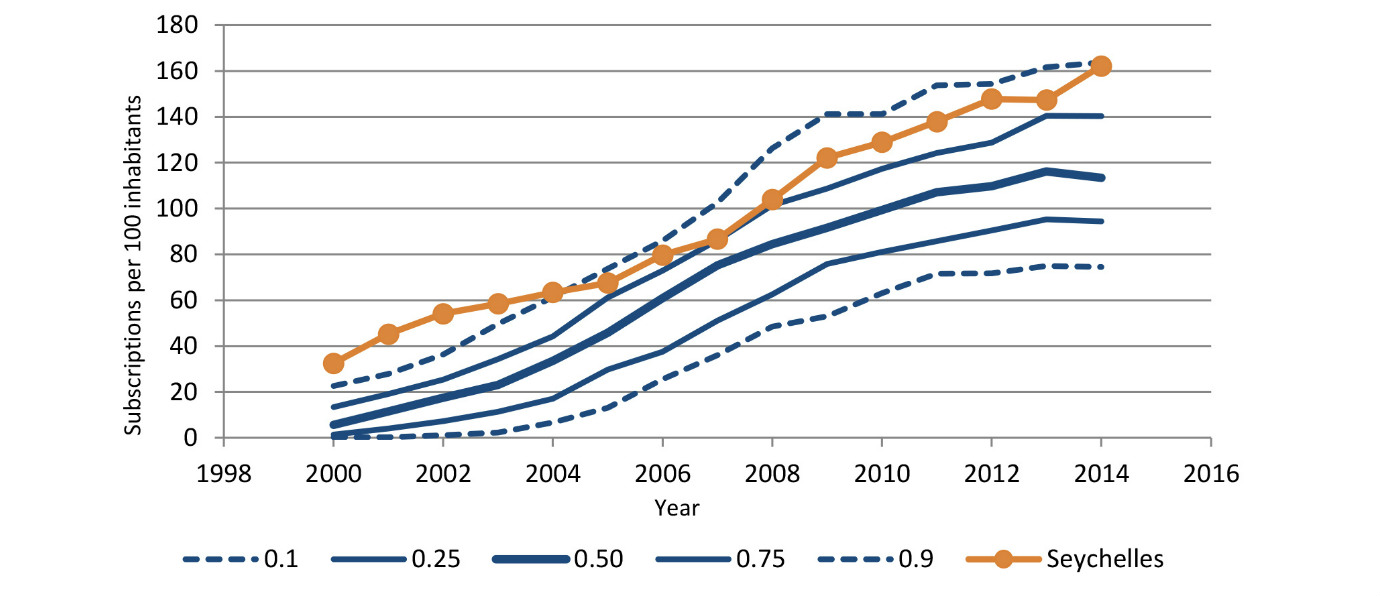
Mobile phone subscriptions per 100 inhabitants in percentiles for 48 upper middle-income countries between 2000 and 2014.

In recent decades, there has been a considerable rise in mobile phone ownership in low- and middle-income countries [9]. Based on data from the International Telecommunication Union [9], Figure 1 highlights the growth of mobile phone subscriptions in 48 upper middle-income countries. Figure 1 illustrates that the rate of mobile subscriptions per 100 inhabitants in 50% of upper middle-income countries increased more than 20-fold between 2000 and 2014. The Seychelles—our study site—consistently ranked in the top 25th percentile of upper middle-income countries throughout this period, reaching 160 mobile subscriptions per 100 inhabitants in 2014 [9]. By 2010, the penetration of mobile phones in the Seychelles had surpassed household ownership rates of landline phones (49%), radio (87%), and television (95%) [10].

Mobile-based interventions are increasingly used in health promotion campaigns providing target-specific SMS that encourage specific behavior changes such as increased fruit and vegetable consumption [11], smoking cessation [12], and adoption of healthy lifestyles [13]. Furthermore, mobile-based approaches have also been increasingly applied to address various aspects of disease prevention such as appointment keeping, medication adherence, medical test results delivery, remote diagnosis, data collection, access to health records, disease tracking, and medical response in emergency situations [7,14-18].

SMS- or email-based programs offer several advantages over traditional mass media for health promotion and disease prevention, as they provide opportunities for interactive 2-way communication [15] and target specific, tailored behavior change communication [16,19]. Such interventions offer the opportunity for dissemination of automated, timely, and target-specific messages, which can be designed to complement or mirror in-person counselling [20,21]. For example, messages can offer tailored advice, behavior tracking, goal setting, encouragement, or personal feedback in different stages of behavior change [22-24]. Many theories focus on the need for health messages to offer predisposing, reinforcing, and enabling components of effective health interventions [25]. Several emerging behavior theories suggest that SMS- or email-based interventions can provide timely health messages that can match the level of an individual’s motivation and his or her ability to act, and therefore facilitate behavior change [26]. While many studies have shown that mobile phone strategies, using either voice or SMS messaging, can encourage behavior change by increasing patient self-efficacy and assisting in chronic disease management [15,20,24,27-29], further research on SMS-based interventions is required to design effective public health interventions using mobile technology.

A major constraint in the success of any mHealth initiative is its ability to reach the target population, as well as its adoption, acceptance, and utility from the users’ perspective [20,30]. Numerous studies have highlighted the potential inequity in access to technology-based services resulting from differences in adoption of new technologies such as mobile ownership or Internet access, a concept known as the digital divide [31-33]. Socioeconomic indicators such as sex, education, and income are a few of the many determinants of this digital divide. While the rapid rise in mobile ownership in low- and middle-income countries is reducing the socioeconomic gap in mobile ownership, there remain important differences in adoption of modern technology, particularly in developing countries [31]. Despite this existing gap, several mobile-based initiatives have been successfully implemented in African countries providing health care services to remote areas [24,27-29,34-37].

To date, mHealth interventions in Africa have mostly focused on human immunodeficiency virus/AIDS, malaria, and maternal and child health [20]. However, evidence in support of these approaches in addressing the burden of NCDs in African countries is growing, mainly aimed at improving patient-provider communication [17]. Recent studies have highlighted the benefits of mHealth interventions for cancer care in rural Cameroon [34], Zambia [35], and Nigeria [36] and diabetes care in Western Kenya [37]. However, a continued challenge with health education interventions is to ensure that exposure to health messages extends to all persons targeted, whether a particular group (eg, persons at risk of NCDs) or the entire population, including persons of low SES or otherwise other sociodemographic characteristics that make them less susceptible to be exposed to a particular mass medium.

Despite the overall rise in mobile penetration in developing countries, it is important to assess the penetration of mobile phones and other electronic devices across different demographic, SES, and health risk categories in order to adequately design SMS- or email-based health education interventions. Using data from a national health survey in 2013–2014 in the Seychelles, we assessed 1) self-reported exposure to programs related to NCDs on national public television and radio during the 12 months preceding the survey, 2) current ownership of mobile phones, smartphones, tablets, or computers and Internet access, and 3) willingness of individuals to receive emails or SMS related to information on NCDs, with a focus on the distribution of these variables across different demographic, SES, and NCD risk groups.

## Methods

We conducted a nationally representative survey (Seychelles Heart Study IV) in 2013–2014 in the Republic of Seychelles, a rapidly developing small island state in the Indian Ocean, east of Kenya. The survey followed the WHO STEPwise approach to surveillance and was approved by the Ministry of Health of the Seychelles following a technical and ethical review [38,39]. The eligible population included a sex- and age-stratified sample of all adults aged 25-64 years of the 3 main islands, based on computerized data of 2010 national population census, which was thereafter regularly updated by civil authorities. The final study sample consisted of 1240 participants, reflecting a participation rate of 73%.

A structured questionnaire was administered face-to-face by trained survey officers. The questionnaire assessed, among other information, sociodemographic characteristics (age, sex, income, education, and occupation), self-reported health behaviors (including physical activity, smoking, and alcohol habits), exposure to programs related to NCDs on national public television or radio in the past 12 months, ownership and use of selected information and communication technology (mobile phone, computer, tablet, and access to email and Internet), and willingness to receive emails or SMS with information related to health.

Body weight and height were measured and body mass index was calculated as weight (in kg) divided by squared height (in meters). Blood pressure was measured and the average of 3 readings was considered. High blood pressure was defined as blood pressure ≥140/90 mmHg or receiving blood pressure treatment. Elevated blood glucose was defined as plasma glucose >6.1 mmol/L or taking antidiabetic medication. Persons at risk of developing NCD included those who were smoking, drank excessive amounts of alcohol, were not physically active, had high body mass index, or had high blood pressure, high cholesterol, or diabetes. Multimedia Appendix 1 lists the variables used in this study and their definitions.

We examined the distribution of outcomes across demographic, SES, and NCD risk groups and evaluated group differences using Wald tests. We used logistic regression analysis to assess the associations between socioeconomic variables and our outcome variables (exposure to traditional mass media, ownership of mobile phone and other electronic devices, access to and use of the Internet, and willingness to receive health-related emails and SMS), adjusting for age, sex, and SES. Estimates for crude and adjusted odds ratios were not markedly different and therefore we show only adjusted estimates. Analyses were weighted to reflect the actual population distribution of persons aged 25–64 years. *P* <.05 was considered significant. Analyses were performed using STATA/SE version 12 software (StataCorp LP).

## Results

Table 1 presents the distribution of demographic, SES, and NCD risk categories in the population. Of the 1240 adults, around half had obtained education beyond obligatory education (ie, beyond the age of 15–16 years, and approximately one-third of adults earned more than 8000 Seychelles rupees per month (~US$ 600), in line with data from the Seychelles National Bureau of Statistics [40]. In the sample of 1240 participants aged 25–64 years, the prevalences were 64.32% (n=798) for overweight or obesity, 59.90% (n=743) for hypertension, 21.36% (n=265) for elevated blood glucose, 20.60% (n=255) for smoking, 25.41% (n=315) for heavy alcohol intake, and 23.16% (n=287) for sedentary lifestyle. All proportions are adjusted to the actual age and sex distributions of the population of Seychelles.

### Mobile Technology Ownership

Table 2 shows the univariate distribution of ownership of electronic devices and Internet access by sex, age, and SES categories and the multivariate odds ratios for the relationships between the outcomes when adjusted for all the variables displayed in the table. Overall, of the 1240 adults aged 25–64 years, 1156 (93.21%) owned a mobile phone. The distribution differed by sex: 1191 women (96.04%) versus 1120 men (90.32%); age group: 1197 aged 25–34 (96.58%) versus 996 aged 55–64 (80.34%) years; and SES: 1228 persons with university education (99.03%) versus 860 persons who did not complete obligatory school (69.35%). Of the 1240 adults, 396 (31.93%) owned a smartphone, 245 (19.75%) used their smartphone to access the Internet, 678 (54.67%) owned a computer or tablet at home, and 477 (38.46%) had Internet access with a personal computer or tablet. The social patterning of these outcomes was generally comparable with that of mobile phone ownership described above. We observed similar age and SES differences in adjusted models, even if these associations were not always statistically significant.

### Exposure to Health Programs on Mass Media

The first 2 columns of Table 3 show the exposure to health programs on NCDs through traditional mass media (limited to health programs from national public radio and television broadcasting company). It is important to note that, in our sample of 1240 adults, 1234 (99.51%) owned a television and 1221 (98.46%) owned a radio. In Table 3 , the reported proportions are univariate, while odds ratios are multivariate, adjusted to other variables. Participants were asked about their exposure to health education programs on national public television or radio on cardiovascular disease, including stroke and heart attack; cardiovascular risk factors such as hypertension, blood cholesterol, or diabetes; and lifestyle habits such as smoking, alcohol drinking, diet, and physical activity. Overall, 1036 of the 1240 participants (83.54%) reported having viewed at least one such program on public television during the past 12 months, while only 740 of the 1240 participants (59.67%) reported having listened to at least one such program on public radio during the same time period.

In multivariate analysis, viewing health-related television programs was higher among women and more mature adults, and there was no clear systematic relationship with SES. Similarly, exposure to health-related programs on public radio was higher among women and older adults, but negatively associated with SES, even though the estimated coefficients were generally not statistically significant.

### Willingness to Receive Health Massages by SMS or Email

The last 2 columns of Table 3 present willingness to receive health-related SMS or email messages. For self-reported willingness to receive health-related messages, 1048 of the 1240 (84.51%) participants expressed willingness to receive such information by SMS compared with only 508 participants (40.96%) by email. In univariate analyses, this proportion was significantly associated with female sex: 1096 women (88.39%) versus 1000 men (80.64%); age: 1128 young adults at age 25–34 (90.96%) versus 808 older adults at age 55–64 (65.21%,) years; and SES: 1031 adults with highest (83.14%) versus 634 adults with lowest education categories (51.12%). When adjusted for all explanatory variables at the same time, willingness to receive SMS or emails related to health continued to be positively associated with female sex, younger age, and higher SES, consistent with results of the univariate analyses.

Table 4 examines the proportion of those with NCD risk factors with the likelihood of exposure to NCD-related health programs on national public media and willingness to receive SMS or emails related to health, when unadjusted and adjusted for age, sex, and SES, in addition to all NCD variables. Univariate analysis showed a higher proportion of those with hypertension and diabetes having watched television or radio programs on health in the past 12 months. However, these differences are no longer significant in a multivariate model when adjusted for SES variables. Findings were similar for willingness to receive health-related SMS or email messages. These findings suggest that when individuals’ variation in age, sex, income, education, and occupation are taken into account, NCD risk factors (ie, hypertension, elevated blood glucose, overweight, smoking, or high alcohol intake) do not significantly increase or decrease their interest in NCD-related interventions via mass media or mHealth.

**Table 1:** Characteristics of the study population (Seychelles, N=1240, 2013–2014).

Population characteristics			No.	%
**Sociodemographic variables**				
	**Sex**		
		Male	621	50.11
		Female	620	49.98
	**Age (years)**				
		25–34	356	28.68
		35–44	362	29.35
		45–54	327	26.36
		55–64	197	15.85
	**Education**				
		Did not complete obligatory	91	7.37
		Completed obligatory	560	45.16
		Vocational	174	14.02
		Polytechnic	344	27.76
		University	72	5.77
	**Annual income (Seychelles rupees)**				
		<3000	106	8.52
		3000–5000	312	25.17
		5000–8000	441	35.53
		8000–15,000	280	22.59
		≥15,000	102	8.20
	**Occupation**				
		Nonqualified laborer	254	20.46
		Semiqualified manual	351	28.28
		Qualified manual	193	15.57
		Semiqualified nonmanual	256	20.64
		Qualified professional	224	18.05
**Clinical variables**					
	**Body mass index (kg/m**^2^ **)**				
		<25 (normal weight)	432	34.82
		25–30 (overweight)	424	34.22
		≥30 (obese)	373	30.10
	**Physical activity**				
		Sedentary	287	23.16
		Moderate	780	62.90
		Active	173	13.95
	**Alcohol consumption**				
		None	524	42.22
		Moderate	401	32.37
		Heavy	315	25.41
	**Smoking status**				
		Current smoker	255	20.60
		Ex-smoker	119	9.57
		Never smoked	866	69.84
	**Hypertension**				
		Negative	497	40.10
		Positive	743	59.90
	**Elevated blood sugar**				
		Negative	975	78.66
		Positive	265	21.36

**Table 2:** Univariate distribution and multivariate odds ratios for ownership of mobile phone, smartphone, computer, or tablet, and Internet access, by sex, age and socioeconomic status (Seychelles, N=1240, 2013–2014).

Population characteristics		Owns a mobile phone		Owns a smartphone		Has access to Internet with smartphone		Owns a personal tablet or computer		Has access to Internet with personal computer or tablet	
		%	aOR^b^	%	aOR	%	aOR	%	aOR	%	aOR
Total		93.2		31.9		19.7		54.3		38.5	
**Sex**											
	Male	90.4	1	28.9	1	18.0	1	51.7	1	38.4	1
	Female	96.1	*6.81* ^c^	34.9	*1.44*	21.4	*1.49*	56.9	*1.52*	38.6	1.09
	*P* value	<.001		.03		.17		.08		.94	
**Age (years)**											
	25–34	96.6	1	51.8	1	33.7	1	63.6	1	47.8	1
	35–44	96.5	0.72	30.8	*0.40*	18.5	*0.43*	62.0	0.93	42.4	0.74
	45–54	93.6	0.61	24.8	*0.34*	13.3	*0.33*	49.5	*0.59*	35.1	*0.60*
	55–64	80.3	*0.27*	9.90	*0.15*	7.10	*0.22*	31.3	*0.37*	20.1	*0.38*
	*P* value	<.001		<.001		<.001		<.001		<.001	
**Education**											
	Did not complete obligatory	69.4	1	6.10	1	4.70	1	17.4	1	10.1	1
	Obligatory	91.8	*1.98*	19.8	1.84	10.00	1.02	38.1	1.31	20.1	0.87
	Vocational	95.9	2.54	43.9	*3.85*	28.7	2.48	58.8	*2.66*	30.1	1.82
	Polytechnic	99.2	*8.65*	45.8	*3.17*	28.3	2.03	80.0	*3.45*	65.3	2.82
	University	99.1	7.70	63.9	*7.42*	51.5	*5.53*	94.5	*6.51*	87.6	*5.64*
	*P* value	<.001		<.001		<.001		<.001		<.001	
**Annual income (Seychelles rupees)**											
	<3000	74.3	1	9.50	1	4.10	1	30.7	1	15.7	1
	3000–5000	90.8	*2.62*	26.7	1.87	16.3	2.14	37.0	0.69	20.0	0.61
	5000–8000	95.1	*5.45*	30.4	1.95	17.0	1.93	50.4	0.86	34.5	0.89
	8000–15,000	98.0	*7.32*	45.1	*2.55*	28.3	2.68	75.0	1.57	59.7	1.45
	≥15,000	98.7	*13.8*	41.4	1.93	34.2	*3.45*	92.1	*6.14*	77.9	*3.07*
	*P* value	<.001		<.001		<.001		<.001		<.001	
**Occupation**											
	Nonqualified laborer	83.0	1	13.3	1	5.00	1	22.5	1	6.90	1
	Semiqualified manual	93.5	*3.54*	29.6	*1.97*	19.1	*3.31*	43.6	*2.15*	30.2	*4.23*
	Qualified manual	92.8	2.25	26.6	1.29	17.7	*2.33*	55.4	*2.47*	34.7	*3.28*
	Semiqualified nonmanual	98.6	*4.84*	36.9	*2.14*	22.5	*3.08*	71.0	*4.26*	51.4	*6.72*
	Qualified professional	98.4	2.31	54.5	*3.13*	35.4	*3.28*	87.2	*5.53*	75.1	*8.32*
	*P* value	<.001		<.001		<.001		<.001		<.001	

^a^Wald test for univariate differences between the first and last categories of each socioeconomic variable.

^b^Multivariate odds ratios are adjusted (aOR) to all socioeconomic variables (sex, age, education, income, and occupation) with the reference categories identified as 1.

^c^Coefficients with *P* <.05 are represented in italics.

**Table 3:** Univariate distribution and multivariate odds ratios for exposure to television or radio programs on NCDs during the past 12 months and willingness to receive health-related SMSc or email, by sex, age and socioeconomic status (Seychelles, N=1240, 2013–2014).

Population characteristic		Viewed a program on television		Listened a program on radio		Would like to receive SMS messages		Would like to receive email messages	
		%	aOR^d^	%	aOR	%	aOR	%	aOR
Total		83.6		59.7		84.5		41.0	
**Sex**									
	Male	79.3	1	57.8	1	80.7	1	36.4	1
	Female	87.8	*1.92* ^e^	61.6	1.08	88.4	*2.73*	45.3	*1.65*
	*P* value	<.001		.2		<.001		.006	
**Age (years)**									
	25–34	70.1	1	43.8	1	91.5	1	49.5	1
	35–44	87.2	*2.76*	55.2	1.66	87.1	*0.65*	43.7	*0.61*
	45–54	90.0	*3.62*	71.7	3.41	86.3	*0.85*	39.9	*0.54*
	55–64	88.9	*3.20*	76.6	3.88	65.2	*0.33*	19.7	*0.26*
	*P* value	<.001		<.001		<.001		<.001	
**Education**									
	Did not complete obligatory	86.0	1	80.0	1	51.1	1	10.3	1
	Obligatory	87.1	*1.27*	62.0	0.72	83.0	2.57	21.5	*0.65*
	Vocational	74.1	0.84	52.7	0.80	90.1	3.63	32.4	1.04
	Polytechnic	81.7	1.09	55.6	0.96	93.4	5.98	67.5	1.56
	University	84.7	*1.47*	51.9	0.79	83.2	3.61	93.3	*4.93*
	*P* value	.85		<.001		<.001		<.001	
**Annual income (Seychelles rupees)**									
	<3000	85.5	1	75.7	1	60.1	1	10.0	1
	3000–5000	85.3	*1.31*	64.5	0.88	84.5	*2.37*	20.6	*0.92*
	5000–8000	82.6	*1.17*	56.7	0.69	87.7	*2.62*	34.7	*1.26*
	8000–15,000	82.9	1.22	55.6	0.69	89.3	2.22	63.3	2.29
	≥15,000	82.3	0.81	52.0	0.46	83.5	1.99	82.8	5.94
	*P* value	.55		<.001		<.001		<.001	
**Occupation**									
	Nonqualified laborer	85.8	1	72.3	1	69.4	1	3.90	1
	Semiqualified manual	83.0	*1.21*	60.2	0.75	88.1	*3.34*	23.6	*5.91*
	Qualified manual	77.3	*0.97*	51.3	0.57	82.5	*1.73*	33.7	*6.46*
	Semiqualified nonmanual	86.0	*1.06*	54.0	0.55	93.5	*3.08*	59.0	*16.7*
	Qualified professional	83.4	0.96	56.7	0.64	87.2	1.30	82.0	25.4
	*P* value	.49		<.001		.001		<.001	

^a^Wald test for univariate differences between the first and last categories of each socioeconomic variable.

^b^NCDs: noncommunicable diseases.

^c^SMS: short message service.

^d^Multivariate odds ratios are adjusted (aOR) to all socioeconomic variables (sex, age, education, income, and occupation) with the reference categories identified as 1.

^e^Coefficients with *P* <.05 are represented in italics.

**Table 4:** Univariate distribution^a^ and multivariate odds ratios for exposure to television or radio programs on NCDs^b^ during the past 12 months and willingness to receive health-related SMS^c^ or email, by NCD risk factor categories (Seychelles, n=1240, 2013–2014).

Population characteristic		Mobile ownership	Viewed a program on television		Listened a program on radio		Would like to receive SMS messages		Would like to receive email messages	
		%	%	aOR	%	aOR	%	aOR	%	aOR
**Smoking**										
	Smoker	83.3	81.3	1	55.8	1	80.2	1	28.0	1
	Ex-smoker	92.2	85.5	0.93	62.6	0.99	84.5	1.31	39.8	1.47
	Never smoked	96.2	83.9	0.82	60.4	1.24	85.8	0.87	44.5	0.96
		<.001	.41		.25		.06		<.001	
**Alcohol intake**										
	None	94.5	84.8	1	62.3	1	84.6	1	40.7	1
	Moderate	94.8	84.3	1.24	58.6	1.05	85.9	1.13	46.2	0.88
	Heavy	89.0	80.3	0.86	56.5	0.91	82.7	0.88	34.5	0.65
	.006	.14		.12		.50		0.13	
**Physical activity**										
	Sedentary	93.6	83.9	1	58.6	1	86.4	1	59.0	1
	Moderate	93.2	84.8	1.09	60.9	1.03	85.2	0.94	39.8	*0.53* ^e^
	Active	92.7	77.0	0.81	55.7	0.99	78.3	0.57	19.3	*0.3*
		.72	.11		.58		.05		<.001	
**Body mass index**										
	<25	89.2	82.0	1	53.0	1	83.0	1	38.3	1
	25–30	95.7	82.5	0.68	61.1	1.15	84.8	1.01	43.3	0.98
	≥ *30*	94.8	86.4	0.7	65.4	1.22	86.0	1.05	41.2	0.98
		.002	.11		<.001		.42		.49	
**Hypertension**										
	Negative	94.9	79.9	1	53.9	1	87.9	1	45.0	1
	Positive	90.7	89.0	1.27	68.3	1.05	79.4	0.74	34.8	0.87
		.003	<.001		<.001		<.001		.002	
**Elevated sugar**										
	Negative	94.8	81.8	1	56.7	1	86.9	1	42.5	1
	Positive	87.2	90.0	1.36	70.7	1.09	75.8	0.79	34.9	1.23
		<.001	.001		<.001		<.001		.002	

^a^Wald test for univariate differences between the first and last categories of each socioeconomic variable.

^b^NCDs: noncommunicable diseases.

^c^SMS: short message service.

^d^Multivariate odds ratios are adjusted (aOR) to all socioeconomic variables (sex, age, education, income, and occupation) with the reference categories identified as 1.

^e^Coefficients with *P* <.05 are represented in italics.

## Discussion

We found a large exposure to programs related to NCDs on national public television in the adult population aged 25–64 years in the Seychelles. This exposure was especially large among women and older persons, with no significant association with SES. On the other hand, exposure to health programs on radio was lower than on that on television, with higher exposure among persons of lower SES than among higher SES. We found that the majority of adults owned a mobile phone, but fewer owned smartphones, computers, or tablets or had Internet access. The willingness to receive health-related SMS was higher in women, younger adults, and those in higher SES. We also found that willingness to receive health-related SMS was not independently related to a person’s NCD risk. Overall, this study highlights the different reach, according to age, sex, and SES, of health messages on NCDs supplied through public mass media programs versus health messages that would be based on SMS or email. In particular, our findings emphasize the presence of a digital divide according to age, sex, and SES despite the large penetration of mobile phone and other new electronic media in the population. This divide does not mean that modern media are inappropriate for health education programs related to NCDs, but it suggests that interventions based on mobile technology should be carefully designed with regard to specific purposes and audiences. Our findings may have relevance for other countries that are similar to the Seychelles, including other small island developing states or some middle-income countries that have achieved rapid socioeconomic development.

The sociodemographic differences in mobile ownership and willingness to receive health-related SMS or email messages, as found in this study, play a key role in ensuring that mHealth initiatives have an equitable reach among the target populations [30]. While SMS-based interventions have a promising potential for disease prevention and health service delivery, such interventions can also potentially exacerbate health inequalities arising from a digital divide [41]. Our findings are consistent with a social pattern in the uptake of health-related information [42].

In addition to a social digital divide, numerous studies have also suggested a sex divide in mobile technology adoption, with greater mobile access among men than among women in most developing countries [33,43,44]. In contrast, we found that more women than men owned a mobile phone or a smartphone. Women were also more willing to receive health-related SMS and were more likely to watch or listen to NCD-related programs on public television and radio during the year under study. These differences may be small in absolute magnitude but can be important when designing health-related public education programs. This sex difference favoring women over men may be partially explained by the relatively high sex equity in Seychelles, for example, the Seychelles ranked second highest out of 52 African countries for sex equity according to the Ibrahim Index [45].

We also found an age-related digital divide, with modern communication technologies being used more often by younger than by older persons. This age-related digital divide was small for ownership of mobile phones but was larger for smartphone ownership, and access to and use of the Internet. Also, fewer older than younger persons were willing to receive SMS or emails related to health. This is consistent with younger adults and persons of higher SES being prone to adopt new technologies [33,46,47].

We found that persons at higher risk of specific NCD conditions were not more likely to watch or listen to NCD-related public television or radio programs, and were not more willing to receive health-related SMS or emails than were persons at lower NCD risk, when adjusted for demographics and SES. It is possible that persons with NCD risk would have shown more interest in SMS-based health programs if they had been fully aware of the many potential benefits of mHealth in general, such as SMS reminders to attend medical visits or to take medications, or health messages tailored to a person’s particular condition.

The finding that the use of mobile communication devices, or willingness to receive health-related SMS or email messages, is higher among younger adults and among persons of higher SES—irrespective of NCD risk—has important public health implications, since NCDs tend to concentrate in persons of older age and in persons of lower SES. The social and age-related digital divides not only stem from different levels of ownership of mobile devices, but also relate to differences in motivation, ability, and skills to use these devices and the related apps. It is likely that the age and socioeconomic digital divides will decrease in the coming generations, which will allow for persons from broader age and SES categories also to benefit from mHealth services. More generally, age, sex, and social differences in the use of mHealth stress the need to carefully design interventions and to address potential equity concerns.

On the other hand, the larger ownership of mobile phones and smartphones, and access to the Internet, as well as the larger acceptance of health-related SMS and emails, among younger adults may be viewed as an advantage when designing SMS-based mHealth initiatives among young adults. In the same line, the frequent use of email and Internet among persons with qualified occupations suggests that this target population could benefit most from Web-based and email health messaging at the work place.

We also found that, despite the overall rise in the use of new media and communication technologies, large proportions of adults continue to listen to and watch health programs on radio and television. This is consistent with a strong commitment from the Seychelles’ health authorities to frequently broadcast radio and television programs on health during the past 25 years. We found that exposure to NCD-related programs on television was fairly uniform across age, sex, and SES groups. Hence, these programs may be useful to educate the public about health issues of general interest, such as raising awareness of NCDs in the general public, emphasizing the importance of good medication adherence, explaining the components of a healthy diet, or stressing the importance of regular blood pressure checks. We also found that programs on NCDs on public radio had a larger audience among older persons and among persons of lower SES, which also corresponds to the population subgroups at higher risk of NCD. Hence, there may be some benefit for continued use of traditional media to broadcast health education programs related to NCDs in developing countries such as the Seychelles.

Several limitations of this study need to be highlighted. The age range was limited to 25–64 years, that is, we excluded both children (when healthy behaviors are ingrained) and the elderly (those at higher risk of NCDs). Also, our questions on willingness to receive health-related email or SMS were not designed to assess the whole range of mHealth services. Similarly, questions on exposure to health programs on traditional media did not assess the impact of these programs on behaviors. Finally, while the findings in our study may possibly extend to a few other rapidly developing small island states or certain upper middle-income countries that have experienced rapid socioeconomic development similar to that in the Seychelles, further research is required to replicate these findings in similar contexts. Strengths of the study include the population-based design, the fairly large sample size, and the assessment of numerous variables related to the use of mobile technology and to the exposure to health programs related to NCDs.

In conclusion, our study offers new evidence on exposure of health-related programs through traditional media and the feasibility and acceptance of mHealth interventions in an upper middle-income country. With a high reach among all groups of the population, national television programs on health appeared to continue to serve as a valuable medium for health promotion and NCD prevention. A large majority of the population owned a mobile phone and were willing to receive health-related SMS messages. However, due to heterogeneous distribution of mobile technology and a digital divide at the time of the survey, mHealth intervention showed the highest potential reach among persons with higher income and education, as well as in younger adults. These finding are important to design and implement mHealth interventions or health programs in the mass media. More generally, this study highlights advantages and disadvantages of traditional mass media versus modern mobile technology for providing health education and the substantial differences in exposure that can occur according to age, sex, and SES. Our findings further emphasize that health education interventions supplied through traditional mass media or through modern mobile technology must be carefully designed in terms of the intended targeted audiences.

## Appendix

Multimedia Appendix 1 Study survey questions and response categories.

## Abbreviations

NCD: Noncommunicable disease
SES: socioeconomic status
SMS: short message service
WHO: World Health Organization
