# Supplementary material for: Potential Reach of mHealth Versus Traditional Mass Media for Prevention of Chronic Diseases: Evidence From a Nationally Representative Survey in a Middle-Income Country in Africa
Source: J Med Internet Res. 2016 May 20;18(5):e114. doi: 10.2196/jmir.5592 (PMC4893150; doi:10.2196/jmir.5592)
Supplement: Multimedia Appendix 1 [file jmir_v18i5e114_app1.pdf]

Appendix 1: Study survey questions and responses categories

	Measuring details	Classification criteria
Socio-demographic variables		
Age groups	Equally distributed age groups	25-34, 35-44, 45-54, 55-64
Education	(Self-reported) What is the highest level of education you have completed?	Did not complete obligatory school<br>Completed obligatory school<br>Attended a vocational school<br>Attended a high-school or technical school<br>Attended a university
Income	(Self-reported) What is your personal earnings (SRP) per month, on average (incl. all allowances, benefits, etc)? (1000 SRP = $78 USD)	1: <3000<br>2: 3000-5000<br>3: 5000-8000<br>4: 8000-15'000<br>5: >15'000
Occupation	(Self-reported) current occupation, categorized by interviewer, or last held job if unemployed.	Non-qualified (labourer)<br>Semi-qualified manual (some training);<br>Qualified manual (trained mechanic, farmer)<br>Semi-qualified non-manual (clerk)<br>Professional/ qualified non-manual (teacher)
NCD variables		
BMI	(Measured) Calculated based on measured weight (kg) divided by height (m) squared and classified according to WHO recommendations [46].	Normal weight (18.5-24.9)<br>Overweight (25.0-29.9)<br>Obese (> 30.0)
Physical activity	(Self-reported) Which of the following best suits your physical activity pattern in relation to doing work in your workplace or at home?	Sedentary: sitting always or most often<br>Moderate: often walking, standing up or carrying light weights<br>Heavy: walking or standing up a lot, or regularly carrying heavy weights
Alcohol consumption	(Self-reported) Averages number of specific drink type consumed per week x its volume in ml x its alcohol in %)/7= grams of alcohol per day<br>(10 grams of alcohol = 1 unit)	Non-drinker: 0 unit /week<br>Moderate drinker (men): 1-21 units/week<br>Moderate drinker (women): 1-14 units/week<br>Heavy drinker(men): > 22 units/week<br>Heavy drinker (women): > 15 units/week
Smoking status	(Self-reported) Smoking category	Current/occasional smoker<br>Ex-smoker<br>Never smoked
Hypertension	(Measured) Average of three blood pressure measurement by a trained nurse and grouped as per cut-off recommendation [47].	Positive: > 140 SBP or >90 DBP<br>Negative: <140 SBP and <90 DBP
Elevated blood Glucose	(Measured)fFasting glucose analysed on capillary blood<br>Recommended cut off [47]	Negative: fasting plasma glucose < 6.1 mg/dl<br>Positive: fasting plasma glucose > 6.1 mg/dl
Mobile technology assessment		
Mobile ownership	Do you have a mobile phone?	Yes or no
Internet on mobile	Can your mobile phone access internet (e.g. smart phone: Iphone, Android, Blackberry, Windows, …)?	Yes or no
Internet use on mobile	Do you use internet with your phone?	Yes or no
Personal computer	Do you have a tablet or computer (your own or that you can use whenever you want outside of work)?	Yes or no
Internet on PC/tablet	Do you use internet with a computer or tablet (outside of work)?	Yes or no
Email	Do you have an email address?	Yes or no
SMS for health	Would you like to receive SMS (text) on your	Yes or no

	phone related to health?	
Email for health	Would you like to receive emails related to health?	Yes or no
Mass media		
TV program on NCD	In the past 12 months, can you remember to have watched a program on cardiovascular disease, smoking, blood pressure, diabetes or diet on SBC TV?	Yes or no
Radio program on NCD	In the past 12 months, can you remember to have listened to a program on health, particularly cardiovascular disease, smoking, blood pressure, diabetes or diet on SBC radio?	Yes or no
